# Retinal Wnt signaling defect in a zebrafish fetal alcohol spectrum disorder model

**DOI:** 10.1371/journal.pone.0201659

**Published:** 2018-08-01

**Authors:** Pooja Muralidharan, Swapnalee Sarmah, James A. Marrs

**Affiliations:** Department of Biology, Indiana University-Purdue University Indianapolis, Indianapolis, Indiana, United States of America; Oregon State University, UNITED STATES

## Abstract

Fetal alcohol spectrum disorder caused by prenatal alcohol exposure includes ocular abnormalities (microphthalmia, photoreceptor dysfunction, cataracts). Zebrafish embryos exposed to ethanol from gastrulation through somitogenesis show severe ocular defects, including microphthalmia and photoreceptor differentiation defects. Ethanol-treated zebrafish had an enlarged ciliary marginal zone (CMZ) relative to the retina size and reduced Müller glial cells (MGCs). Ethanol exposure produced immature photoreceptors with increased proliferation, indicating cell cycle exit failure. Signaling mechanisms in the CMZ were affected by embryonic ethanol exposure, including Wnt signaling in the CMZ, Notch signaling and *neurod* gene expression. Retinoic acid or folic acid co-supplementation with ethanol rescued Wnt signaling and retinal differentiation. Activating Wnt signaling using GSK3 inhibitor (LSN 2105786; Eli Lilly and Co.) restored retinal cell differentiation pathways. Ethanol exposed embryos were treated with Wnt agonist, which rescued Wnt-active cells in the CMZ, Notch-active cells in the retina, proliferation, and photoreceptor terminal differentiation. Our results illustrate the critical role of Wnt signaling in ethanol-induced retinal defects.

## Introduction

Vertebrate retina has six main cell types including, retinal ganglion cells (RGCs), bipolar cells, amacrine cells, horizontal cells, photoreceptors, and the major glial cell type, Müller glial cells (MGCs). Retinal cell types are born progressively as development proceeds: commencing with RGCs and ending with bipolar cells, rods, and MGCs. Retinal precursors exit cell cycle periodically and sequentially, as developmental signaling pathways regulate neurogenesis and gliogenesis [1–3]. In zebrafish, retinal growth continues radially throughout the life of the fish. Multipotent retinal stem cells reside in the ciliary marginal zone (CMZ) [4, 5]. The CMZ generates retinal cell types, including MGCs. MGCs in the inner nuclear layer (INL) produce rod precursors, which rapidly divide to form a neurogenic cluster, migrate to outer nuclear layer (ONL) and terminally differentiate to form rod photoreceptors [6–8].

The CMZ stem and progenitor cells determine the growth and differentiation of the retina. Based on cell cycle and proneural gene marker expression patterns, the CMZ is roughly divided into three major zones: (i) peripheral CMZ with low expression of cell cycle activators; (ii) middle CMZ with high expression of Cyclin D1 and high proliferation; and (iii) central CMZ with high cell cycle inhibitor (*p57*^*kip2*^) and proneural gene expression [9, 10]. Evidence from zebrafish, *Xenopus*, and chick retinal models show that self-renewal and differentiation of multipotent retinal stem cells in the CMZ is tightly regulated by Wnt, Sox2 and Notch signaling [11–20]. In the peripheral CMZ, canonical Wnt signaling can inhibit neural retinal differentiation and maintain a proliferative, undifferentiated state through Notch by blocking proneural activity [12, 14, 20]. Wnt signaling activation inhibits GSK3β, leading to β-catenin translocation into nucleus, which binds TCF/LEF transcription factors and activates transcription of target genes. Notch activation causes nuclear translocation of notch-intracellular domain that, on binding with other co-factors, leads to basic helix-loop-helix (bHLH) transcriptional repressor expression, Hairy-enhancer of split (*hes*) genes. *hes* genes expression can actively repress neurogenesis. Notch and Sox2 signaling coordinate to determine proneural gene expression levels. As stem cell differentiation progresses into middle and central CMZ with reduced Notch activity, these mitotic precursor cells express proneural and other transcription factors, exit the cell cycle and undergo terminal differentiation. Following activation of proneural genes, other signaling pathways such as Shh, retinoic acid (RA), Fgf, and Bmp regulate proliferation, differentiation and patterning [21–25]. For example, rod photoreceptor precursors and progenitors, express differentiation factors (such as, *neurod*, *crx*, and *rx1*), migrate to the ONL, express rod-specific transcription factors, and finally terminally differentiate into rods [26]. Later born glial cells require Notch activation, which promotes MGC differentiation [27].

Retinal development is very sensitive to teratogen exposure [28]. Previous studies using zebrafish showed that embryonic ethanol exposure produced severe retinal cell differentiation defects [29–31], including reduced photoreceptor differentiation and optic nerve hypoplasia [31]. Cell differentiation initiation occurred with the same timing, but a small eye phenotype was evident by 48 hpf, when the central retinal neurogenesis is close to completion, suggesting that cells arising from the CMZ are affected [29, 31–33]. Specific regions of the retina, including the CMZ, INL and ONL showed increased proliferation in the ethanol treated fish [31]. Ethanol-induced cell cycle exit failure was found in the retina and central nervous system. [34].

Ethanol-mediated developmental toxicity defects were identified, including oxidative stress, nutrient metabolism, epigenetic modification, and gene expression regulation [35–39]. Many studies also showed rescue experiments with antioxidants and nutritional compounds [34, 40–44]. Studies on ethanol-induced craniofacial and cardiac defects show rescue by RA and folic acid (FA) co-supplementation [41, 42, 45, 46]. RA and FA co-supplementation with ethanol during early gastrulation and somitogenesis also rescued eye defects, such as photoreceptor differentiation defects and optic nerve hypoplasia [31]. Ethanol competitively inhibits RA biosynthesis [47, 48], which could inhibit RA-signaling dependent processes in the retina including, photoreceptor differentiation and retinal patterning. FA is a critical factor involved in one-carbon metabolism and nucleic acid synthesis [49]. Ethanol exposure was shown to reduce maternal FA absorption leading to a wide range of defects due to ethanol-induced FA deficiency [50]. The cellular and molecular mechanisms underlying rescue by RA and FA supplementation are unclear.

Here, ethanol effects on retinal stem cell compartments and retina developmental signaling pathways were examined to understand the genesis of persistent ethanol-induced retinal defects. Ethanol exposure affected the CMZ structure, Wnt and Notch signaling. Experiments demonstrate that Wnt signaling in the retina is a crucial mechanism underlying ethanol-induced retinal cell differentiation defects, highlighting a potential therapeutic target for this devastating birth defect.

## Materials and methods

### Zebrafish husbandry

This work was conducted according to guidelines and supervision of the Indiana University Policy on Animal Care and Use. The IUPUI School of Science Institutional Animal Care and Use Committee (IACUC) reviewed and approved this study. Anesthesia was performed using tricaine, and euthanasia used anesthetic overdose with physical assurance. Zebrafish (*Danio rerio;* TL and AB wild type strains; transgenic line that expresses *Tg(nrd*:*eGFP)* [51]; *Tg(TP1bglob*:*eGFP)* (referred to as *Tg(TP1*:*GFP)*) [52]; *Tg(TP1*:*mCherry)* [52]; *Tg(gfap*:*GFP)mi2001* (referred to as *Tg(gfap*:*GFP)*) [53]; and *Tg(Tcf/Lef-miniP*:*dGFP)*^*isi01*^(referred to as *Tg(Tcf/Lef-miniP*:*dGFP)* [54] transgenic lines) were raised and housed under standard laboratory conditions [55] in accordance with Indiana University Policy on Animal Care and Use. *Tg(nrd*:*eGFP)* expresses eGFP under the *neurod* gene promoter. *Tg(TP1bglob*:*eGFP)* (referred to as *Tg(TP1*:*GFP)*) and *Tg(TP1*:*mCherry)* express color variants of GFP under the regulation of repeated sequence elements that are activated by Notch intracellular domain binding, that is, gene expression is activated by Notch receptor signaling. *Tg(gfap*:*GFP)mi2001* (referred to as *Tg(gfap*:*GFP)* expresses GFP under the *gfap* gene promoter. *Tg(Tcf/Lef-miniP*:*dGFP)*^*isi01*^(referred to as *Tg(Tcf/Lef-miniP*:*dGFP)* expresses GFP under the regulation of repeated sequence elements that bind Tcf/Lef DNA binding factors that complex with β-catenin to activate gene expression. The embryos were treated with 1-phenyl-2-thiourea (0.003%) from 6 hpf (shield) onward in order to prevent melanogenesis. Potential secondary effects of 1-phenyl-2-thiourea in larval to juvenile stage transition will need additional investigation [56].

### Embryo treatments

Zebrafish embryos were exposed to ethanol by incubation in embryo medium containing 100 mM (0.6% v/v) or 150 mM (0.9% v/v) ethanol (referred to as 100 EtOH and 150 EtOH respectively) from 2–24 hpf as previously described in [31, 44]. The embryos were supplemented with 1 nM RA or 75 μM FA from 2–24 hpf were performed as previously described in [44]. Zebrafish were treated with 350 nM and 500 nM GSK3β inhibitor compound (LSN 2105786; Eli Lilly and Co.) from 32–48 hpf and 48–72 hpf. These fish were then kept in control embryo medium until desired stage was reached and fixed using 4% Paraformaldehyde (PFA) in phosphate buffered saline (PBS).

### Microscopy

Brightfield images for histological and ISH sections were captured using Axiovision camera ICc1 mounted on the Zeiss observer Z1 (10X 0.3 NA; 20X 0.8 NA). Confocal images of whole mount immunofluorescence staining and FISH were collected using Zeiss Observer Z1 (40X 1.1 NA W). All zebrafish were deyolked and imaged from the ventral side. Z-sections analyzed always included the optic nerve for consistency. Confocal imaging allowed us to collect images that were not saturating the photomultiplier tube detector, and thus linear fluorescence intensity is linear within the entire dynamic range.

### Immunofluorescence

Fixed zebrafish were used for whole mount immunostaining as previously described [57] using primary antibodies against Alcama (zn-5, 1:200; ZIRC), phospho-Histone 3 (1:500; Millipore), HuC/D (1:1000; Sigma), zpr-1 (1:1000; ZIRC). Alexa-Fluor 488- conjugated anti-mouse and anti-rabbit (1:200), Alexa-Fluor 555-conjugated goat-anti mouse (1:200), Alexa-Fluor 647-conjugated goat-anti mouse (1:200), and Texas-Red conjugated anti rabbit (1:100) secondary antibodies (Molecular Probes) were used. Nuclear staining was performed using TO-PRO-3 iodide at 1:1000 dilution for 1 hour. Eyes were imaged to produce optical sections using confocal microscopy.

### In situ hybridization (ISH)

Whole mount ISH was performed as previously described [58] using digoxigenin-labeled (dig-labeled) riboprobes for genes *rx1 (retinal homeobox gene 1*), *axin2* (Addgene), *her6* (*hes1b*, *hairy-related 6*, generously provided by Dr. Pamela Raymond). Dig-labeled riboprobes were synthesized using a Dig RNA labeling kit (Roche). Stained embryos were mounted for histological analysis (see below). Brightfield images were collected.

### Fluorescence in situ hybridization (FISH)

Whole mount FISH was performed using dig-labeled riboprobes for the genes, *cdkn1c* (*p57* ^*kip2*^, *cyclin-dependent kinase inhibitor 1c*; T7-protomter binding site containing primers; Table 1). Dig-labeled riboprobes were synthesized using Dig RNA labeling kit. Zebrafish were fixed overnight in 4% PFA in PBS at 4°C, washed in PBS containing 0.1% Tween 20 (PBST), dehydrated stepwise to methanol, and stored at -20°C. After rehydration, fish were permeabilized with proteinase K, re-fixed and washed. They were incubated in hybridization buffer for 2 hours and the riboprobe was added for overnight incubation at 70°C. Following stepwise washing to 2X SSC, 0.05X SSC and PBT, the fish were placed in blocking solution (1X maleic acid buffer, 2% BSA and 2% normal goat serum) for 2 hours at room temperature. The fish were incubated in Anti-Dig POD (1:1000; Roche) in blocking solution overnight at 4°C. Finally, the fish were washed with PBS and developed with tyramide signal amplification kit. Alexa fluor-488 labeled tyramide (1:100) with hydrogen peroxide was added to the fish for 60–90 minutes at room temperature. The fish were washed three times for 10 minutes with PBST, and imaged using a confocal microscope [59].

**Table 1:** Primer sequences used in the study. Underlined sequence indicates T7/T3 promoter binding consensus sequence.

Primer name	Sequence (5’-3’)	Reference
RT-*kip2*-F	CTTCAGTCCTCAGAAACAGACGGAAG	[84]
RT-*kip2*-R	CATCCGCTCTGCAGATAAACACAGGTG	
*kip2* –T7F	tgaattgTAATACGACTCACTATAGGGcggataaagtacaaaacaagagagctc	
*kip2*- T3R	aagctcgaAATTAACCCTCACTAAAGggcactttgattcaaaggtacaacgtgagc	
*rx1*- F	GGACCAGGATTCGTTGCTCA	[85]
*rx1*- R	ATCCCTAAGGGGTGGCAGAT	
*axin2*- F	GGACACTTCAAGGAACAACTAC	[86]
*axin2*- R	CCTCATACATTGGCAGAACTG	
*rsp15-F*	CAGAGGTGTGGACCTGGACCAGC	[44]
*rsp15-R*	CGGGCAGGATGACCATGTCTCTC	

### Histology

Four and 18 dpf (days post-fertilization) zebrafish larvae were fixed with 4% PFA and embedded in JB-4^TM^ resin (Polysciences) for plastic sections which was performed at the Indiana University Histology Core. Five μm ventral sections were obtained using Leica RM2265 microtome and were stained with hematoxylin and eosin (H&E). Whole mount ISH zebrafish were also similarly mounted in JB-4^TM^ resin for 5 μm plastic sections. Brightfield images were collected.

### Quantitative PCR analysis

Total RNA was extracted from 72 hpf ethanol-treated and untreated retinas using TRIzol reagent (Sigma). One microgram of total RNA was reverse transcribed to cDNA using M-MLV reverse transcriptase (Promega, Madison). Each PCR reaction was performed with 1 microliter of cDNA using Power SYBR Green PCR mix (Applied Biosystems, Foster City). Primer sets used are listed in Table 1. Independent experiments in triplicates were performed using *rsp15* endogenous control, which has been shown in our previous studies to remain unchanged after ethanol treatment. Thermal cycling was done using the 7300 Real Time PCR system (Applied Biosystems). Relative expression was calculated as described [60]. Fold changes in gene expression was calculated using comparative CT method (ΔΔCT).

### Image analyses and cell counting

H&E stained histological sections containing optic nerve were used for image analyses. CMZ area and retinal area were measured using Image J software (NIH software). CMZ area was demarcated to the lamination in the retina. *Tg(TP1*:*mCherry)*, Notch-reporter transgenic line was used to perform cell counts of nuclear-localized mCherry-positive cells representing the Notch-active cells. A single optic nerve-containing optical section was used in 72 hpf control and ethanol treated larvae. The measurements of total zpr-1 intensity per retina in the ONL and cell proliferation counts per retina were performed on zpr-1 and phosphoH3 stained fish, respectively, as described earlier [31].

### Statistical analysis

Unpaired two-tailed student’s t-test was used for comparisons between control and ethanol treated groups using GraphPad software (GraphPad). One-way ANOVA and post hoc Tukey HSD for individual comparisons were used for analyses in rescue experiments.

## Results

In this study, zebrafish embryos were treated from 2–24 hpf with 100 and 150 mM ethanol (0.6 and 0.9% v/v, referred to as 100E and 150E, respectively). Previous studies show that zebrafish larvae treated with 166.67 mM (1% v/v) from 6–24 hpf, have an equilibrated ethanol concentration inside the embryo that ranges from 40–58 mM [61]. This exposure regimen is a chronic ethanol exposure model during a developmental period corresponding to approximately the first trimester of human development. Ethanol exposed embryos showed severe retinal cell differentiation defects [31]. These previous studies showed that ethanol delays retina lamination, and thus, developmental delay could be a factor in this model.

### Ethanol-induced expansion of the CMZ

**Fig 1:**
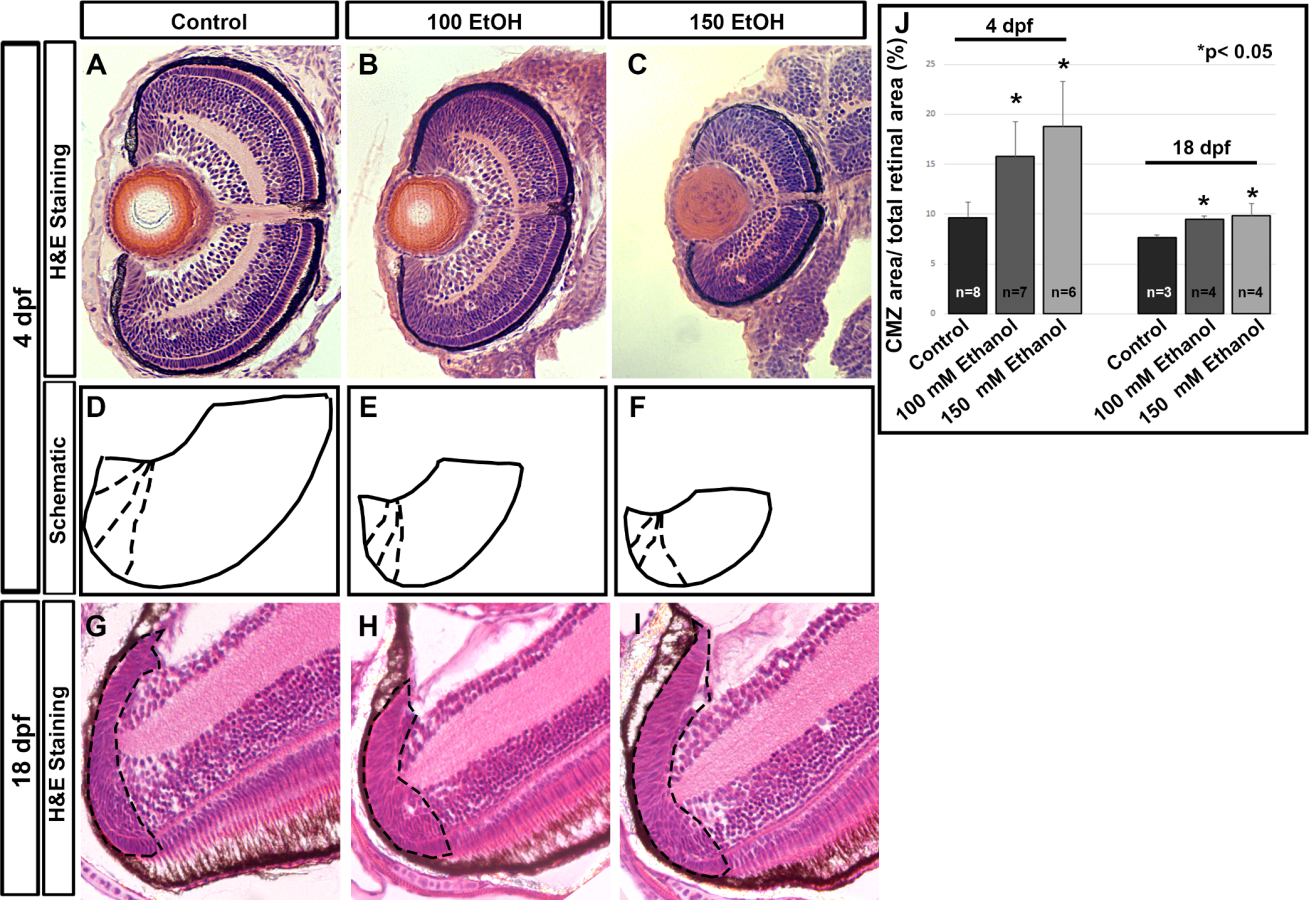
Early ethanol exposure altered CMZ composition. (A-C) H&E staining of control (A), 100 mM ethanol (B), and 150 mM ethanol treated larvae (C) show persistent defects at 4 dpf. (D-F) Schematic drawn of the ventral half of the retina in proportion to the histology sections at 4 dpf (A-C). The solid line indicates the retinal area ending at the optic nerve and the outer most layer of the retina. Dashed lines indicate the various parts of the CMZ as can be distinguished by the nuclear morphology. (G-I) H&E staining of control (G), 100 mM ethanol (H), and 150 mM ethanol treated fish (I) show persistent defects at 18 dpf. The ethanol treated fish (H, I) showing slightly expanded CMZ due to ethanol exposure. Black dashed lines indicate the CMZ area, which is demarcated based on retinal lamination and cell morphology. (J) Ratio of CMZ area to the retinal area showing an increase in CMZ in proportion to the retina persistently at 4 and 18 dpf. Images show rostral at top, lateral at left. Error bars indicate standard deviation. ‘*’ indicates statistical significance in comparison to control embryos (p<0.05).

Histology at 4 dpf and 18 dpf indicated a persistent microphthalmia in ethanol treated larvae (Fig 1A–1C and 1G–1I). At these later stages, ethanol treated fish also show persistent changes in the CMZ relative to controls. In the 4 dpf retinas, CMZ cells in ethanol treated larvae showed distinct nuclear morphological differences as compared to controls. The compact nature of the nuclei in the control CMZ was not as evident in the ethanol treated larvae. Consequently, CMZ area in proportion to the retinal area showed a significant increase in comparison to control fish (Fig 1J). This is more evident in the schematic of the ventral half of the retina drawn to scale, showing an expansion of the CMZ in ethanol exposed larvae relative to control neural retina at 4 dpf (Fig 1D–1F). When the absolute CMZ size in control versus ethanol treated fish was compared, there is not a significant change, raising the possibility of a developmental delay mechanism. An alternative hypothesis is that the ethanol-induced changes in relative CMZ/retina proportions (increased relative size of CMZ and reduced relative size of retina) are due to stem cell dysfunction, producing the microphthalmia phenotype.

**Fig 2:**
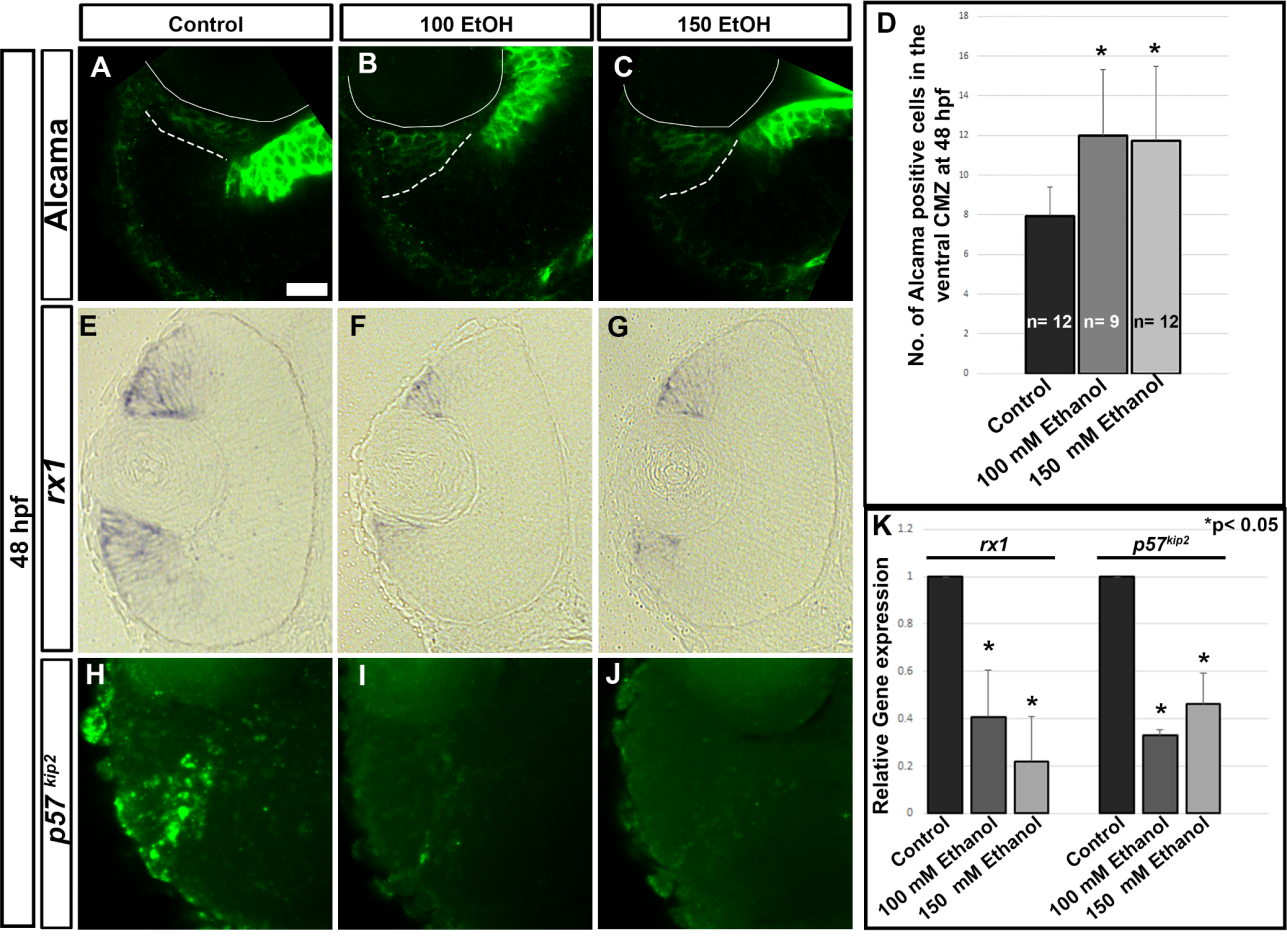
Cellular changes caused in the CMZ due to ethanol exposure. (A-C) Alcama staining of ethanol treated embryos (B, C) showed expansion in comparison to control embryos (A) at 48 hpf in peripheral CMZ. Solid while lines indicate the lens and dashed lines indicate expression domain of Alcama. (D) Quantification of Alcama positive cells in the ventral CMZ at 48 hpf. The number of Alcama positive cells in a single optic nerve containing confocal section of the retina was counted. (E-G) *rx1* ISH sections showed reduced expression after ethanol treatment in peripheral CMZ. (H-J) *p57*^*kip2*^ FISH experiment showed reduced expression in central CMZ. Images show rostral at top, lateral at left. (K) qPCR showed reduced transcript levels of *rx1* and *p57*^*kip2*^ genes at 72 hpf after ethanol treatment in comparison to control retinas. Fold changes in gene expression was calculated using comparative CT method (ΔΔCT). Error bars indicate standard deviation. ‘*’ indicates statistical significance in comparison to control embryos (p<0.05). Scale bar = 10 μm.

To study the CMZ, marker expression patterns were examined using immunostaining, ISH, and qPCR. At 48 hpf, Alcama, a neuroepithelial marker that labels multipotent retinal stem cells and RGCs in the retina showed an expanded expression domain in the peripheral CMZ after ethanol treatment (Fig 2A–2C). Quantification of the number of Alcama-positive cells in the CMZ showed a significant increase in ethanol treated embryos in comparison to controls (Fig 2D). *rx1* expression, which labels multipotent retinal stem cell populations in the CMZ at 48 hpf, was analyzed using ISH (Fig 2E–2G). Ethanol exposure reduced *rx1* expression domain at 48 hpf (Fig 2E–2G). Cyclin dependent kinase inhibitor (*cdkn1c* or *p57*^*kip2*^; a cell cycle exit marker) expression in control embryos is restricted to central CMZ, which was greatly reduced in size in ethanol treated fish (Fig 2H–2J). Expression levels for *rx1* and *p57*^*kip2*^ measured using qPCR was significantly decreased at 72 hpf in ethanol treated retinas (Fig 2K).

### Ethanol exposure affects terminal differentiation

**Fig 3:**
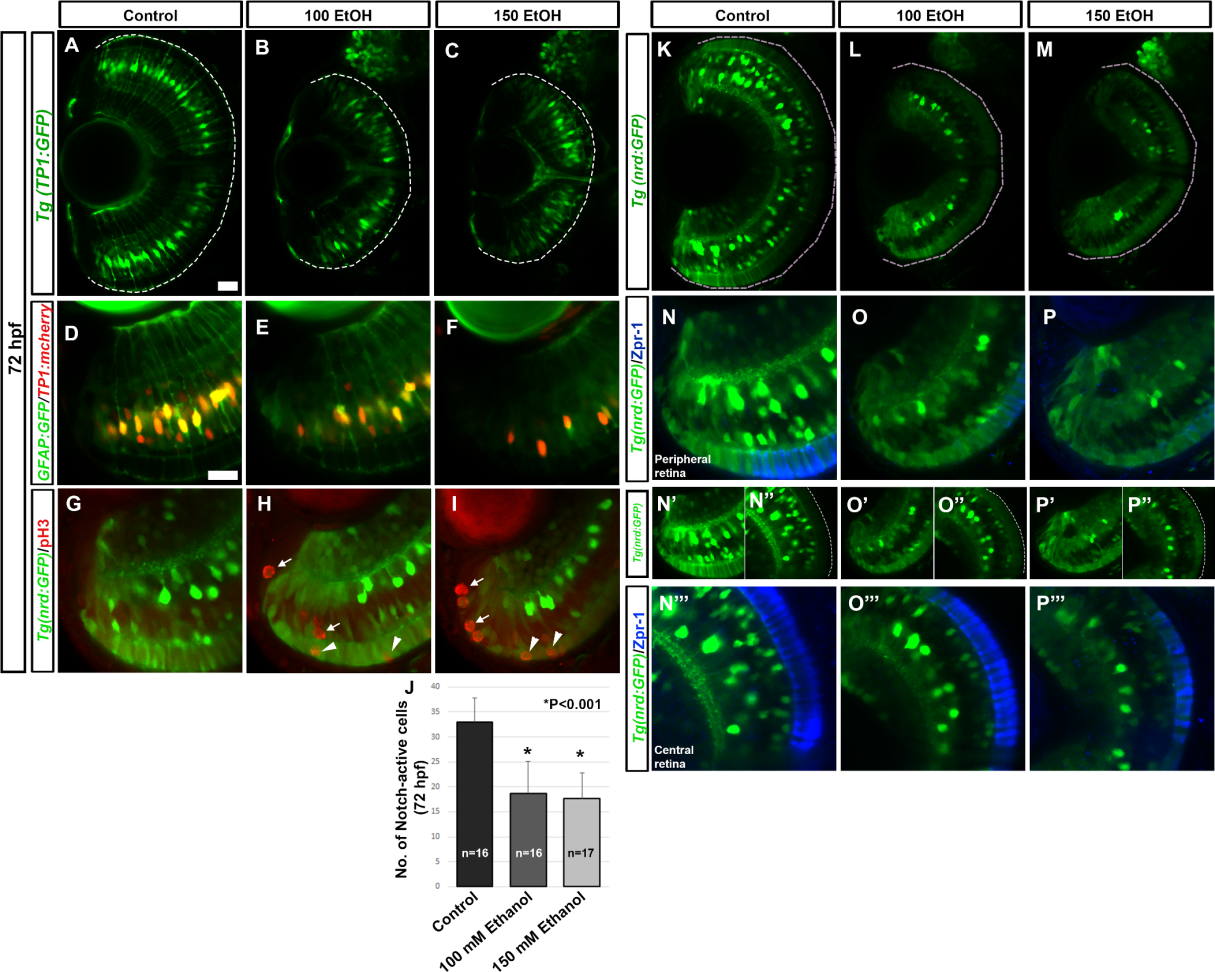
Effect of ethanol on MGC and central retina precursor cell populations. (A-C) Transgenic line *Tg(TP1*:*GFP)* showed reduced Notch-active cells after ethanol exposure at 72 hpf. (D-F) Double transgenic line *Tg(gfap*:*GFP)* and *Tg(TP1*:*mCherry)* showed reduced Notch- and GFAP- double positive cells after ethanol exposure at 72 hpf. (G-I) PhosphoH3 immunostaining on *Tg(nrd*:*GFP)* larvae showed an increased number of mitotically active cells in the ONL and INL, many of which co-labeled with *neurod*- positive cells. (J) Quantification of Notch-active cells per optic-nerve containing optical section of the retina using *Tg(TP1*:*mCherry)* showed a significant decrease after ethanol treatment in comparison to control fish. A single optic nerve-containing optical section of 72 hpf control and ethanol treated larvae was used. (K-M) *Tg(nrd*:*GFP)* larvae showed reduced expression of *neurod* after ethanol exposure at 72 hpf. (N-P”‘) zpr-1 immunostaining on *Tg(nrd*:*GFP)* larvae showed fewer zpr-1 positive cells in the peripheral retina after ethanol exposure.(N-P). In the central retina, zpr-1-positive cells were double labeled with *neurod* after ethanol exposure (N”‘-P”‘) compared to *Tg(nrd*:*GFP)* expression showing expanded *neurod* expression in the ONL (N’, N”, O’, O”, P’, P”). White dashed lines indicate retinal pigmented epithelium (RPE). Images show rostral at top, lateral at left. Error bars indicate standard deviation. ‘*’ indicates statistical significance in comparison to control embryos (p<0.05). Scale bar = 20 μm for panels A-C and K-M. Scale bar = 10 μm for panels D-P”‘.

Notch signaling regulates neuronal vs glial fates during retinal development [60–62]. Differentiating MGCs also express GFAP, Glial fibrillary acidic protein [63]. Using a transgenic reporter line, *Tg(TP1*:*mCherry)*, Notch activity was examined after ethanol exposure (Fig 3A–3C). Ethanol exposure caused reduced Notch activity at 72 hpf in comparison to controls. The number of Notch-active cells at 72 hpf was significantly fewer after ethanol treatment in comparison to controls (Fig 3J). To examine the ethanol-induced MGC defects, double transgenic line, *Tg(TP1*:*mCherry)*, expressed in Notch active cells, and *Tg(gfap*:*GFP)*, expressing GFP under *gfap* promoter were used. At 72 hpf, GFP expression and active Notch signaling was seen in central retina of control larvae (Fig 3D). However, after ethanol exposure, at 72 hpf, retinas showed fewer GFP positive and fewer Notch-active cells, indicating reduced MGC differentiation (Fig 3E and 3F).

Rod photoreceptor lineage arises from asymmetric MGC division in the INL, which migrate to the ONL [26, 62]. These rod precursors are proliferative and cells of the cone and rod lineage express *rx1*, *crx* and *neurod* [26, 62]. To examine rod photoreceptor precursor proliferation, *Tg(nrd*:*GFP)*, larvae expressing GFP under *neuord* promoter, were stained using phospho-Histone-3 (H3) antibody. Ethanol treated larvae showed an increased number of double positive cells in the INL and ONL, indicating more proliferative neural precursor cells (Fig 3G–3I).

The proneural gene *neurod* expression also showed differences after ethanol exposure (Fig 3K–3M). At 72 hpf, bright *Tg(nrd*:*GFP)* expressing cells in the INL, which are a subset of differentiated amacrine cells, could be seen in control larvae [62]. Retinas from ethanol treated larvae showed fewer *neurod*-reporter positive cells in the INL (Fig 3K–3M). At 72 hpf, *neurod*-reporter positive cells in the ONL were expanded in ethanol treated larvae compared to controls. In control larvae, nascent and immature photoreceptor cells in the ONL, in the central CMZ showed *neurod* expression, which transitioned into *neurod* and zpr-1 (detecting red-green double cones) double positive cells and finally mature photoreceptors were zpr-1 positive with minimal *neurod* expression (Fig 3N–3N”‘). Unlike the control larvae, ethanol treated larvae showed cells in the central CMZ and ONL that co-labeled for *neurod* and zpr-1, indicative of poorly differentiated cells, which were immature or nascent cones (Fig 3O–3O”‘ and 3P–3P”‘). These results indicate that retinal cells remained in a precursor state and were incompletely differentiated after embryonic ethanol exposure.

### Ethanol exposure disrupted Wnt signaling and other differentiation pathways

Wnt signaling plays a crucial role in the maintenance of retinal stem cell population [16]. Wnt regulates the decision between stem cell self-renewal and differentiation [63]. Exit from the stem cell compartment leads to proliferative growth in the transit-amplifying compartment until mitosis ceases upon terminal differentiation.

**Fig 4:**
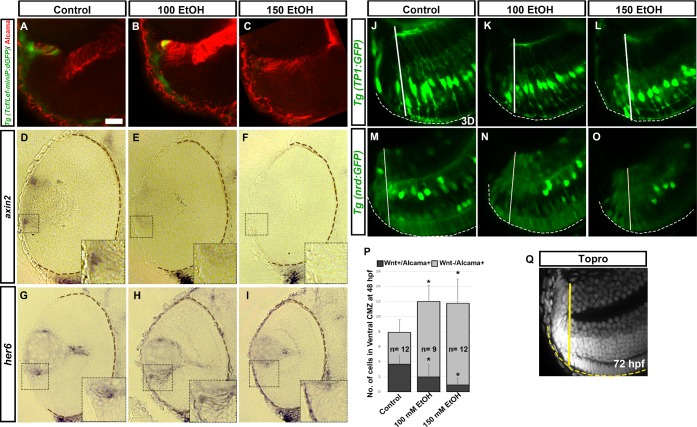
Effect of ethanol exposure on Wnt signaling, and Notch and proneural gene expression. (A-C) *Tg(Tcf/Lef-miniP*:*dGFP)* fish showed Wnt-active cells (green) in the peripheral CMZ labeled with Alcama (red). (D-F) *axin2* ISH showed decreased *axin2* expression in peripheral CMZ after ethanol exposure. (G-I) *her6* ISH sections showed an expansion of *her6* expression in the CMZ after ethanol treatment at 48hpf. Boxed regions in ISH sections highlighting the peripheral retina were magnified in the insets for each image. (J-L) 3D rendition of *Tg(TP1*:*GFP)* fish showed expanded Notch-active cells in the CMZ. (M-O) Central CMZ region of *Tg(nrd*:*GFP)* fish showed increased *neurod*-positive cells after ethanol treatment at 72 hpf. (P) Quantification of Wnt+/Alcama+ and Wnt-/Alcama + cells in the peripheral CMZ. Solid lines demarcate the CMZ from the neural retina based on retinal lamination using TO-PRO-3 staining as indicated (Q, yellow). Dashed lines (white, brown and yellow) indicate RPE of the retina. Numbers of cells were counted in a single optic-nerve containing confocal optical section in the ventral CMZ. Images show rostral at top, lateral at left. Error bars indicate standard deviation. ‘*’ indicates statistical significance in comparison to control embryos (p<0.05). Scale bar = 10 μm for panels A-C and J-O.

Ethanol effects on Wnt, Notch and proneural (NeuroD) signaling pathways involved in retinal stem cell maintenance and cell differentiation were examined using transgenic reporter lines (see Materials and Methods), including *Tg(Tcf/Lef-miniP*:*dGFP)*, Wnt activity-reporter line; *Tg(TP1*:*GFP)*, *Tg(TP1*:*mCherry)*, Notch activity-reporter lines; and *Tg (nrd*:*GFP)*, proneural gene expression transgenic line. Wnt-signaling reporter expression was not detected at 18 hpf (data not shown). Ethanol treatment from 2–24 hpf showed minimal difference in Wnt-active cells in the retina at 24 hpf (data not shown). At 48 hpf, peripheral retina (presumptive CMZ) contains Wnt-active cells in untreated retinas. Most Wnt-positive cells are also Alcama-positive. However, few cells are Wnt-negative and Alcama-positive (Fig 4A). After ethanol exposure, fewer Wnt-positive cells were detected in the CMZ (Fig 4B and 4C). Not only were there fewer Wnt and Alcama double positive cells in ethanol-treated embryos, but the total number of Alcama-positive cells in the CMZ was significantly higher than control embryos (Fig 4P).

Wnt signaling in the peripheral retina was also detected by *axin2* ISH, which is a transcriptional target of Wnt signaling. In control fish, peripheral CMZ showed *axin2* expression (Fig 4D). However, this *axin2* expression was reduced after ethanol treatment (Fig 4E and 4F). *axin2* gene expression levels in the retina using qPCR showed a significant decrease (p<0.05) after ethanol treatment (100 mM ethanol and 150 mM ethanol-treated fish showed 0.294 and 0.562-fold change respectively). These data show that ethanol exposure led to reduced Wnt activity, and an expanded neuroepithelial, immature compartment in the peripheral CMZ.

Our working model suggests that Notch signaling is downstream of Wnt signaling in the CMZ. Retinas from ethanol treated fish showed reduced Notch activity (Fig 3A–3C). In the central CMZ, ethanol treated fish showed increased number of Notch-active cells (Fig 4J–4L). *her6*, a downstream target of Notch signaling, was expressed in the CMZ in a compact layer in the control fish was expanded in 100 mM ethanol treated embryos (Fig 4G). It also appeared to be greatly reduced after 150 mM ethanol treatment. Similar to Notch signaling, *neurod* expression showed an expansion in the central CMZ of the ethanol treated fish further demonstrating proneural and immature nature of these cells (Fig 4M–4O). TO-PRO-3 stained image of the CMZ at 72 hpf was used to demarcate the CMZ area, the unlaminated portion of the peripheral retina (Fig 4Q).

### RA and FA co-supplementation rescued Wnt signaling in the CMZ compartment

**Fig 5:**
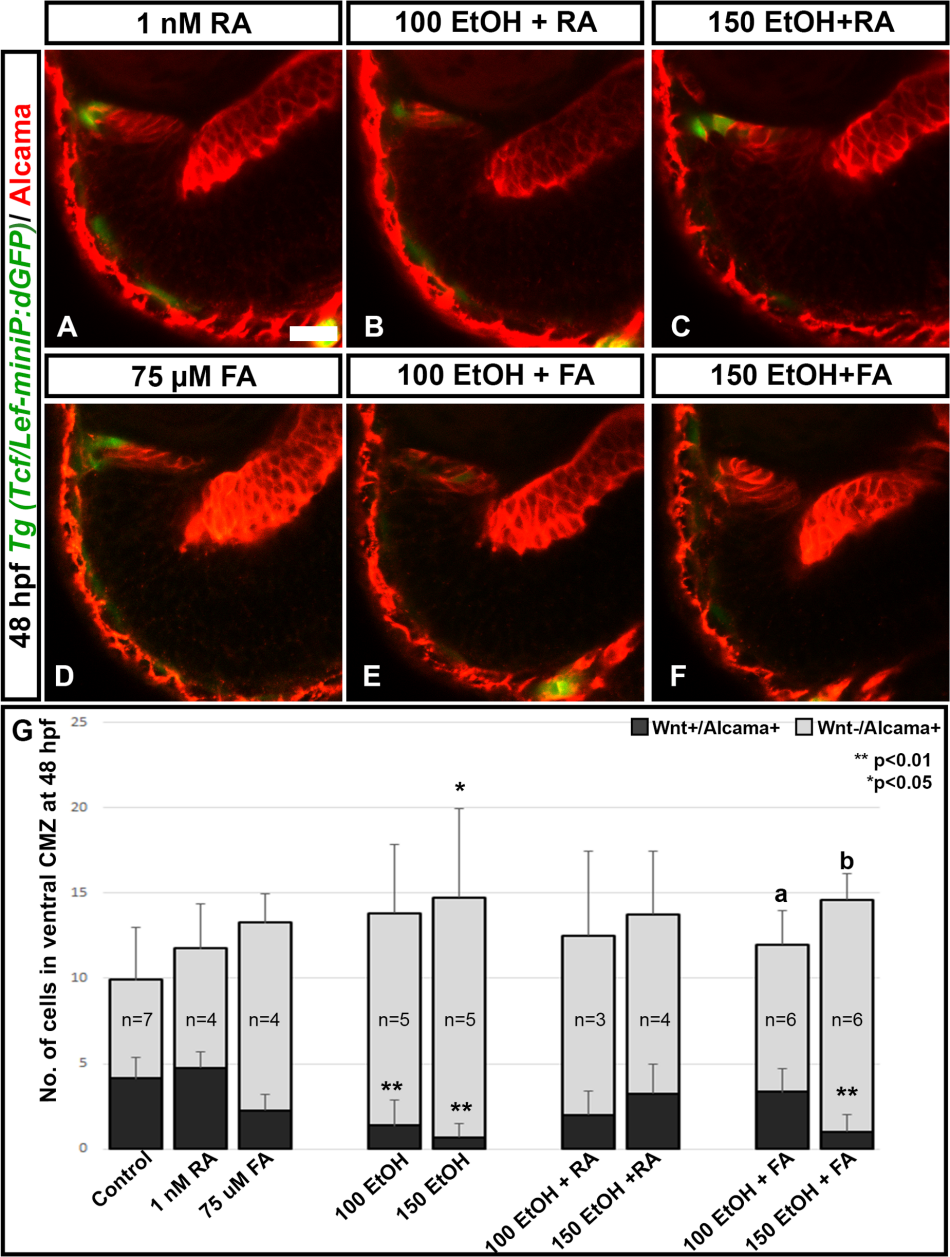
RA and FA co-supplementation rescue Wnt signaling. Alcama staining on *Tg(Tcf/lef-miniP*:*dGFP)* fish showed that ethanol treatment reduced the number of Wnt+/Alcama+ cells in the peripheral CMZ. Control and ethanol treated embryos in this experiment appeared identical to those shown in Fig 4A–4C and were left out for brevity. (A-F) RA co-supplementation could rescue Wnt+/Alcama+ cells (A-C); FA co-supplementation could also rescue Wnt+/Alcama+ cells in the CMZ particularly in 100 mM Ethanol + FA treated fish (D-F). (G) Quantification of Wnt+/Alcama+ cells in the peripheral CMZ showed significant reduction after ethanol treatment, which was rescued by RA and FA co-supplementation. Numbers of cells were counted in a single optic-nerve containing confocal optical section in the ventral CMZ. Images show rostral at top, lateral at left. Error bars indicate standard deviation. ‘**’ indicates statistical significance in comparison to control embryos (p<0.01). ‘*’ indicates statistical significance in comparison to control embryos (p<0.05). ‘a’ indicates statistical significance in comparison to 100 mM ethanol embryos (p<0.05). ‘b’ indicates statistical significance in comparison to 150 mM ethanol embryos (p<0.05). Scale bar = 10 μm for panels A-F.

Previous studies showed that RA and FA co-supplementation with ethanol rescues eye phenotypes produced by ethanol exposure [31]. If Wnt signaling is important for retina differentiation, then these rescue treatments will restore Wnt activity in the CMZ. Embryos co-supplemented with 1 nM RA or 75 μM FA with ethanol from 2–24 hpf were tested for various markers for the CMZ and precursor cells. Staining with Alcama antibody showed that ethanol exposure caused expanded Alcama expression domain, which could be rescued by RA and FA co-supplementation (Fig 4A–4C, Fig 5A–5F). In order to examine the effect of RA or FA treatments on Wnt signaling in the CMZ, *Tg(Tcf/lef-miniP*:*dGFP)* embryos were co-supplemented with RA or FA and ethanol. Both RA and FA co-supplementation could rescue Wnt-active cells in the peripheral CMZ (Fig 5A–5F). RA and FA co-supplementation rescued the reduced number of Wnt and Alcama double positive cells in the peripheral CMZ (Fig 5G). However, 150 mM ethanol and FA treated embryos did not show strong rescue (Fig 5F and 5G). The number of Wnt-negative and Alcama- positive cells showed a significant increase after ethanol treatment which was also rescued by RA and FA co-supplementation (Fig 5G).

**Fig 6:**
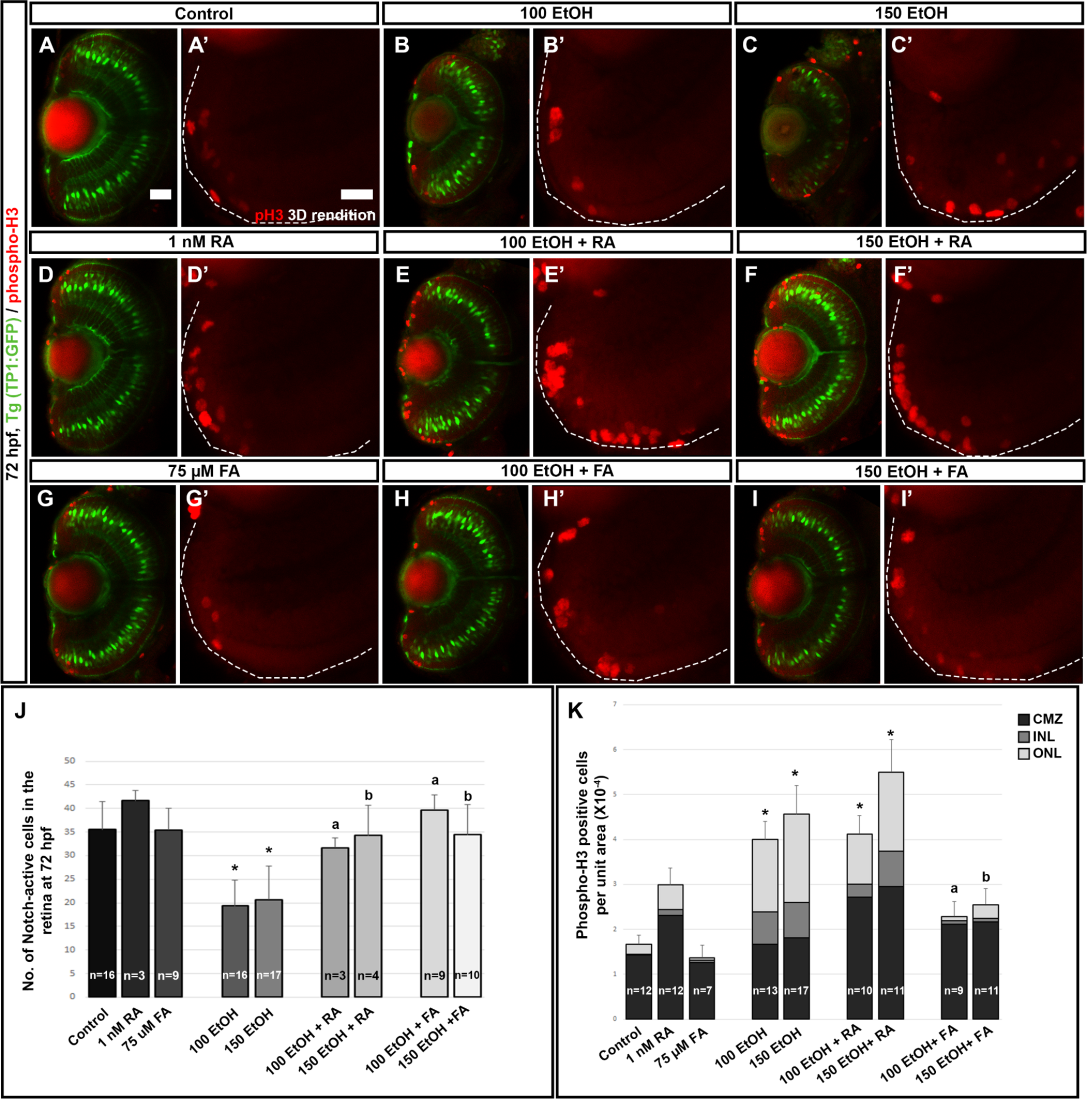
RA, but not FA, co-supplementation induces proliferation. (A-I) PhosphoH3 (red) staining of *Tg(TP1*:*gfp)*, (green cells) larvae showed that ethanol exposure induced proliferation in the CMZ, INL and ONL. (A’-I’) 3D rendition of phosphoH3 staining in the peripheral retinal region showed the increase in proliferation in ethanol treated larvae (B’, C’) which was increased after RA treatment (D’,E’, F’) and rescued after FA co-supplementation (G’, H’, I’). (J) Quantification of mCherry-positive (Notch-active cells) in the retina showed a significant rescue of notch signaling after both RA and FA co-supplementation. (K) Quantification of phosphoH3-positive cells per unit area at 72 hpf showed statistically significant increase in phosphoH3-positive cells in after ethanol treatment which was rescued after FA co-supplementation. Numbers of cells were counted in a single optic-nerve containing confocal optical section in the ventral CMZ. White dashed lines indicate RPE of the retina. Images show rostral at top, lateral at left. Error bars indicate standard deviation. ‘*’ indicates statistical significance in comparison to control embryos (p<0.05). ‘a’ indicates statistical significance in comparison to 100 mM ethanol embryos (p<0.05). ‘b’ indicates statistical significance in comparison to 150 mM ethanol embryos (p<0.05). Scale bar = 20 μm for panels A-I. Scale bar = 10 μm for A’-I’.

Retinas from ethanol treated fish also showed significantly fewer Notch active cells at 72 hpf (Fig 3J). RA or FA co-supplementation with ethanol using Notch reporter *Tg(TP1*:*GFP)* showed rescue of the Notch-active cells to control levels in the retina (Fig 6A–6J). Cell proliferation was increased in ethanol-treated larvae at 72 hpf within the CMZ, ONL and INL of the retina (Fig 6A’–6C’). RA rescue treatment examined at 72 hpf showed a significant increase in cell proliferation over the ethanol treated larvae particularly in the CMZ (Fig 6D’–6F’). Supplementation of RA alone produced a significant increase in cell proliferation in the retina, particularly in the CMZ retinal cells (Fig 6K). In contrast, FA co-supplementation produced a rescue of the ethanol-induced an increase in cell proliferation (Fig 6G–6I’). FA co-supplementation could rescue the ONL and INL cell proliferation levels, but the number of mitotic cells in the CMZ remained similar to ethanol treated retinas (Fig 6K). These findings illustrate that different mechanisms underlie RA and FA rescue. These data also indicate that exogenous RA induced proliferation of the precursor and progenitor cell populations in the central retina and CMZ irrespective of ethanol exposure.

### Wnt activation after ethanol exposure restores downstream signaling and differentiation

**Fig 7:**
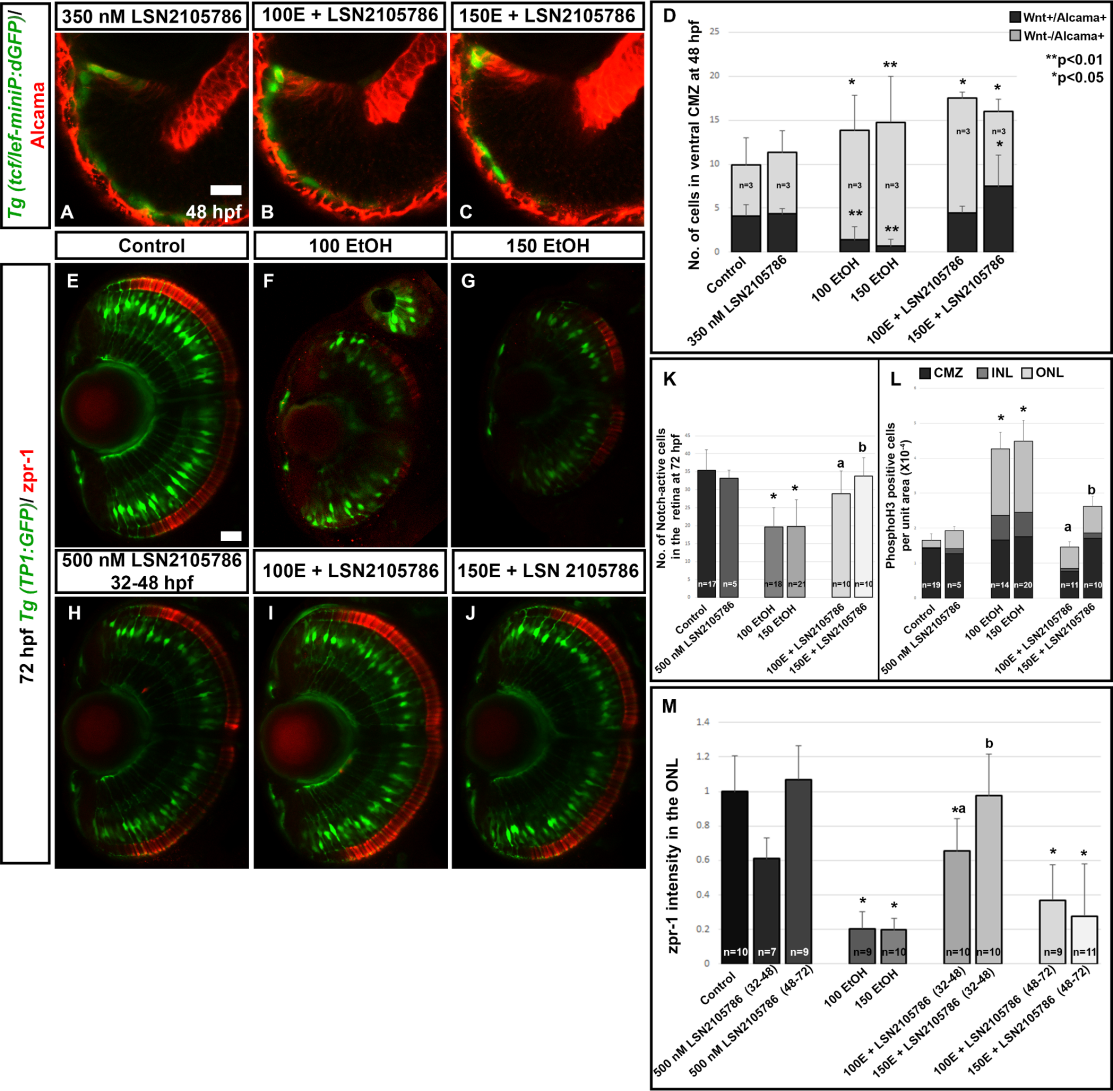
Wnt agonist treatment rescues ethanol-induced retinal cell differentiation defects. (A-C) Treatment with 350 nM GSK3β inhibitor (LSN 2105786) showed a rescue of Wnt+ cells in the peripheral CMZ, which were reduced after ethanol treatment. Control and ethanol treated embryos in this experiment appeared identical to those shown in Fig 4A–4C and were left out for brevity. (D) Quantification of Wnt+/Alcama+ and Wnt-/Alcama+ cells in the peripheral CMZ. (E-J) Treatment with LSN 2105786 on *Tg(TP1*:*GFP)* fish showed rescue of Notch signaling after LSN 2105786 treatment. Ethanol treated fish show reduced notch-activity (green) and photoreceptor terminal differentiation marker expression (zpr-1, red), which was restored by LSN 2105786 treatment. (K) Quantification of mCherry-positive, Notch-active cells per optic nerve containing optical section, in the retina showed a significant rescue of notch signaling after LSN 2105786 treatment. (L) Quantification of phosphoH3-positive cells in the retina showed a significant rescue of cell proliferation response after LSN 2105786 treatment at 72 hpf. (M) Quantification of total zpr-1 intensity in the ONL of the retina showed a significant decrease in photoreceptor marker expression after ethanol treatment (2–24 hpf) and subsequent rescue by LSN 2105786 treatment from 32–48 hpf. 100E+ LSN 2105786, and 150E+ LSN 2105786 indicates 100 mM and 150 mM ethanol exposed embryos treated with LSN 2105786, respectively. Images show rostral at top, lateral at left. Error bars indicate standard deviation. ‘**’ indicates statistical significance in comparison to control embryos (p<0.01). ‘*’ indicates statistical significance in comparison to control embryos (p<0.05). ‘a’ indicates statistical significance in comparison to 100 mM ethanol embryos (p<0.05). ‘b’ indicates statistical significance in comparison to 150 mM ethanol embryos (p<0.05). Scale bar = 10 μm for panels A-C. Scale bar = 10 μm for panels E-J.

Due to the peripheral location of Wnt signaling cells and the cells being a minority in the stem cell compartment, we hypothesize that Wnt signaling defects induced by ethanol exposure are responsible for persistent retina defects. To test this hypothesis, ethanol treated and untreated fish were exposed to GSK3β inhibitor, Wnt signaling agonist (LSN 2105786) and tested for rescue of Notch signaling, proliferation, and photoreceptor differentiation. Since the difference in Wnt signaling was seen after 24 hpf, embryos were treated with 350 and 500 nM LSN 2105786 from 32–48 hpf. At 48 hpf, LSN2105786 treatment produced a rescue of the Wnt activity in the CMZ (Fig 4A–4C; Fig 7A–7C). Quantification of cells in the CMZ showed that ethanol exposure reduced Wnt and Alcama double positive cells and increased Wnt-negative and Alcama-positive cells. LSN 2105786 treatment rescued the Wnt and Alcama-positive cells but did not rescue the Wnt-negative/Alcama-positive cells to control levels (Fig 7D). Perhaps more developmental time or time with Wnt agonist would be needed to fully restore the balance of cell populations in CMZ.

Notch-active cells were examined using *Tg(TP1*:*GFP)* and *Tg(Tp1*:*mCherry)* to count cells at 72 hpf. These experiments showed significantly fewer Notch-active cells in ethanol treated larvae, and Notch-active cell numbers was rescued to control levels by 500 nM LSN 2105786 treatment (Fig 7E–7J; Fig 7K). Embryos treated with LSN 2105786 from 32–48 hpf after ethanol treatment from 2–24 hpf also showed rescue of the proliferation response to control levels, reducing cell proliferation seen in ethanol treated larvae at 72 hpf in the CMZ, ONL and INL (Fig 7L).

Ethanol exposure caused severe photoreceptor differentiation defects at 72 hpf, which was quantified by comparing zpr-1 staining, the red-green double cones with control larvae [29]. To examine the effects of Wnt signaling activation on photoreceptor differentiation, embryos were treated with ethanol from 2–24 hpf, and then, Wnt agonist treatment occurred from 32–48 hpf or 48–72 hpf. Treatment with 500 nM LSN 2105786 from 32–48 hpf, showed a significant rescue of zpr-1 expression intensity in the ONL to control levels. However, later LSN 2105786 treatment from 48–72 hpf, showed only a slight trend towards rescue (Fig 7E–7J; Fig 7M). Perhaps more developmental time or time with Wnt agonist would be needed to restore full photoreceptor differentiation. Together these data show that restoring Wnt signaling rescued Notch signaling and photoreceptor differentiation in ethanol treated larvae.

## Discussion

FASD is a devastating, preventable disorder affecting 1–5% of the US population [64–66]. Ocular defects are frequently associated with FASD defects seen in human patients. Up to 90% of children suffering from fetal alcohol syndrome (FAS) have ocular manifestations [67]. Zebrafish is an excellent model to study retinal development and FASD because timing of ethanol exposure and other treatments can be controlled, and retinal developmental events are well characterized and conserved between vertebrates. Studying retinal defects not only addresses the ocular defects seen in FASD patients but also provides molecular insights into central nervous system defects. Many key signaling pathway disruptions are implicated in FASD. These include but are not limited to RA, epigenetic modifications, and oxidative stress mediated deficits [32, 35–39, 41, 42, 46, 68–71].

Our data shows a persistent defect in the zebrafish retina up to 18 dpf larvae. In order to examine defects, the retinal CMZ was studied. The CMZ was expanded in size, and the CMZ sub-domains were altered by ethanol treatment. Expanded retinal neuroepithelial cell marker expression domain was shown by Alcama staining [72]. Alcama is a cell adhesion molecule and is expressed in hematopoietic, neuronal and other stem cell populations [73]. Although its role in peripheral CMZ of the retina has not been evaluated, it is involved in maintenance of the stem cell niche in hematopoietic and stromal cells [73]. Expanded Alcama expression in ethanol-exposed fish may produce changes in cell adhesion in the peripheral CMZ. Other markers for CMZ cells were affected, including *rx1*, a retinal multipotent stem cell marker.

Another important stem cell population in the neural retina are the MGCs, the radial glia of the retina, which are late born cells that can divide and give rise to rod photoreceptors [26, 74]. Numbers of these cells are greatly reduced in ethanol-exposed larvae in comparison to untreated controls. Notch-Delta signaling regulates MGC specification and differentiation [27]. Notch-active and GFAP-positive MGCs in the central retina are reduced after ethanol treatment, suggesting that defective MGC specification and differentiation in ethanol-exposed larvae are due to Notch signaling defects.

Clinical and experimental studies have shown reduced photoreceptors in FASD patients and various animal systems [29, 30, 32, 34, 75–78]. Our previous study showed that after ethanol treatment, cells were present in the photoreceptor layer, but these cells express lower levels of terminal differentiation markers [31]. Photoreceptor precursors, in the INL and ONL, and terminally differentiated nascent photoreceptors in the ONL, express specific set of transcription factors, including *neurod*, *rx1* and *crx* [26]. To determine the differentiation status of these ONL cells, we used *neurod* and *rx1* markers identifying photoreceptor precursor and progenitor populations, showing more photoreceptor precursor cells and immature photoreceptors in the ONL of ethanol treated larvae. Thus, fewer cells in the ethanol treated fish undergo terminal differentiation and maturation, and the cells present in the ONL of these fish are in a precursor state. Co-labeling with phosphoH3 showed increased number of these cells are mitotically active, indicating cell cycle exit failure. These cells are in photoreceptor lineage but fail to exit the cell cycle. These precursors may lack the cues that normally facilitate their differentiation into mature and functional photoreceptors.

Studies on *Xenopus* retina show that Wnt signaling through Sox2 and Notch modulate proneural gene expression which determine progression from progenitor to differentiated state of cells [12, 19]. Evidence in zebrafish suggests that this mechanism may be conserved [17, 20]. Neuronal differentiation is promoted by: Wnt signaling in peripheral CMZ for stem cell maintenance; increased Sox2 and Notch, which inhibits neurogenesis and promotes gliogenesis; and increased proneural gene expression promotes neuronal differentiation. Closely examining specific signaling pathways governing retinal cell differentiation process showed decreased Wnt and Notch signaling.

Ethanol exposure reduced Wnt activity and *axin2* expression in the peripheral CMZ in our study. Increased GSK3β and consequent cell death has been reported in ethanol treated fetal cerebral cortical neurons [70]. Furthermore, studies in FASD rat models showed increased activation of GSK3β and reduced canonical Wnt and Notch signaling in chronic prenatal ethanol exposed cerebellum [79]. Ethanol-induced noncanonical Wnt signaling disruption has been reported in neural crest cell death [80], and treatment with lithium chloride, a GSK3β inhibitor, protected against ethanol-induced neurotoxicity [80, 81]. Since many molecular pathways underlying retinal development also overlap with CNS development, ethanol-induced reduction in Wnt signaling in the retina agrees with the previous studies in the CNS development. Reduced Wnt signaling may lead to reduced downstream signaling pathway activation, including Notch and *neurod*, which is supported by our rescue experiments using a Wnt agonist (GSK3β inhibitor).

In zebrafish, Wnt signaling hyperstimulation, during specific developmental time windows, led to reduced differentiation and increased proliferation in the retina, and conversely, reduced Wnt signaling led to increased retinal differentiation and reduced proliferation [16]. Ethanol exposure caused reduced Wnt signaling, reduced differentiation and increased proliferation. One possible mechanism for this is that ethanol-induced Wnt signaling defect may produce more progenitor cells in the middle and central CMZ, leading to an increased size of the transit amplifying compartment and thus, increased proliferation. This effect may also explain the expansion of Notch-active and *neurod*-positive cells in the central CMZ of ethanol treated fish as compared to controls. Furthermore, ethanol-induced disruption of other signaling pathways, including RA, may lead to cell cycle exit failure and reduced terminal differentiation [31].

Central retina cells derived from the retinal neuroblasts, which are specified during retinal morphogenesis and not derived from the CMZ, showed ethanol-induced defects. Wnt agonist also rescues central retina, suggesting that there are additional targets of Wnt agonist in addition to the CMZ. Wnt signaling disruption is likely to be only one of the pathways affected by ethanol exposure. Additional studies dissecting the developmental pathways are needed to understand the complete genesis of retinal defects.

Several studies have shown effects of nutrient compound supplementation on ethanol-induced defects [36, 41, 42, 44, 82]. Our previous experiments showed RA and FA supplementation rescues ethanol-induced retinal defects, particularly, retinal photoreceptor cell differentiation and optic nerve development [31]. However, specific differences were identified in RA and FA rescue effects. Although RA and FA co-supplementation could rescue photoreceptor differentiation defects, RA supplementation from 24–48 hpf or 48–72 hpf, after ethanol treatment (2–24 hpf), could restore photoreceptor differentiation but FA post-treatment did not rescue photoreceptor defects. RA signaling is involved in the maintaining the balance of rods, red-green cones, and blue and UV cones in the retina [21, 24]. Studies on zebrafish also show that exogenous RA treatment could induce retinal cell proliferation at 72 hpf [83]. A difference was seen in proliferation response of the cells of RA versus FA treated fish. RA co-supplementation, but not FA co-supplementation, showed increased proliferation in the CMZ, in comparison to larvae only treated with ethanol. Distinguishing differentiation versus proliferation effects of RA in the retina and in our FASD model will require additional investigation.

Ethanol effects on the peripheral CMZ compartment reveals a defect in the stem cell compartment that can lead to persistent retinal defects. Reduced Wnt signaling in this compartment and consequent reduction of Notch activity may underlie many ethanol-induced retinal cell differentiation defects. Restoration of the Wnt-active cells, using GSK3β inhibitor treatment, RA, and FA co-supplementation, restored Notch-activity and terminal differentiation. Additional research examining specific members of the signaling pathways and lineage tracing will provide detailed mechanistic insight in ethanol-induced retinal defects and help identify therapeutic targets for FASD.
